# Low Functional network integrity in cognitively unimpaired and MCI subjects with depressive symptoms: results from a multi-center fMRI study

**DOI:** 10.1038/s41398-024-02891-2

**Published:** 2024-04-05

**Authors:** Gabor Csukly, László Tombor, Zoltan Hidasi, Eva Csibri, Máté Fullajtár, Zsolt Huszár, Vanda Koszovácz, Orsolya Lányi, Edit Vass, Boróka Koleszár, István Kóbor, Katalin Farkas, Viktoria Rosenfeld, Dalida Borbála Berente, Gergo Bolla, Mate Kiss, Anita Kamondi, Andras Attila Horvath

**Affiliations:** 1 Department of Psychiatry and Psychotherapy, Semmelweis University, Budapest, Hungary; 2 Neurocognitive Research Center, Budapest, National Institute of Mental Health, Neurology, and Neurosurgery, Budapest, Hungary; 3 Medical Imaging Centre, Semmelweis University, Budapest, Hungary; 4 Department of Measurement and Information Systems, University of Technology and Economics, Budapest, Hungary; 5 Siemens Healthcare, Budapest, Hungary; 6 Department of Neurology, Semmelweis University, Budapest, Hungary; 7 Department of Anatomy Histology and Embryology, Semmelweis University, Budapest, Hungary

**Keywords:** Depression, Diagnostic markers

## Abstract

Evidence suggests that depressive symptomatology is a consequence of network dysfunction rather than lesion pathology. We studied whole-brain functional connectivity using a Minimum Spanning Tree as a graph-theoretical approach. Furthermore, we examined functional connectivity in the Default Mode Network, the Frontolimbic Network (FLN), the Salience Network, and the Cognitive Control Network. All 183 elderly subjects underwent a comprehensive neuropsychological evaluation and a 3 Tesla brain MRI scan. To assess the potential presence of depressive symptoms, the 13-item version of the Beck Depression Inventory (BDI) or the Geriatric Depression Scale (GDS) was utilized. Participants were assigned into three groups based on their cognitive status: amnestic mild cognitive impairment (MCI), non-amnestic MCI, and healthy controls. Regarding affective symptoms, subjects were categorized into depressed and non-depressed groups. An increased mean eccentricity and network diameter were found in patients with depressive symptoms relative to non-depressed ones, and both measures showed correlations with depressive symptom severity. In patients with depressive symptoms, a functional hypoconnectivity was detected between the Anterior Cingulate Cortex (ACC) and the right amygdala in the FLN, which impairment correlated with depressive symptom severity. While no structural difference was found in subjects with depressive symptoms, the volume of the hippocampus and the thickness of the precuneus and the entorhinal cortex were decreased in subjects with MCI, especially in amnestic MCI. The increase in eccentricity and diameter indicates a more path-like functional network configuration that may lead to an impaired functional integration in depression, a possible cause of depressive symptomatology in the elderly.

## Introduction

### Depression in elderly

Depressive symptoms and late-life depression frequently impair the quality of life of older adults. It affects overall well-being and can make it challenging for older adults to engage in daily activities and maintain social connections. Late-life depression is often underdiagnosed and undertreated. Many older adults may not seek help because they attribute depressive symptoms to normal aging or other physical ailments. According to previous epidemiology studies, the frequency of Late-Life Depression (LLD) varies between 0.9% to 9.4% in private households and 14% to 42% in institutions [1].

Furthermore, almost 50% of subjects with subthreshold or subclinical depression who do not meet the criteria for MDD but show symptoms of depression convert to major depression disorder (MDD) in 18 months. They report function disabilities similar to patients with MDD [2]. Depressive symptoms have significant societal and economic implications. Older adults experiencing depressive symptoms or major depression have a higher mortality rate [3, 4], higher prevalence of physical comorbidities [5, 6], poorer quality of life [7, 8]. They may require additional support services, including mental health professionals, caregivers, and community resources. The association between depression and cognitive decline is well-established [9, 10]. Late-life depression also presents an opportunity for intervention. With appropriate recognition, diagnosis, and treatment, the symptoms of depression can be effectively managed and alleviated, although treatment resistance is prevalent [11, 12]. Early intervention can prevent worsening depressive symptoms, reduce disability, enhance cognitive function, and improve overall health outcomes in older adults [13–15].

### Functional network connectivity in MCI and elderly with depressive symptoms

Depression in older adults is a disease with complex etiology. There is evidence that the factors involved in its development differ from the etiological factors of major depression at a young age, which warrants a separate discussion. The potential association of depression with neurocognitive disorders highlights the prominent role of neurobiological factors in its pathogenesis. Current evidence suggests that depressive symptomatology is a consequence of network dysfunction rather than lesion pathology [16–18]. Combining structural and functional MRI studies is an appropriate method to investigate neural network dysfunction. There is literature evidence that the cortical thickness of certain cortical areas or the volume of specific subcortical structures in LLD differs from that of healthy subjects. In addition, disruption of anatomical connections between these centers has structural or functional correlates with LLD.

The relationship between depression and cognitive decline seems bidirectional, as depression is a risk factor for vascular conditions and Alzheimer’s Disease [19–21]. At the same time, depression can also be an early symptom of cognitive decline [22]. Therefore, the co-examination of MCI and depressive symptomatology on network functioning is a topical scientific issue. While MCI is a result of lesion pathology, accumulating evidence suggests that depressive symptomatology is a consequence of network dysfunction. Based on previous studies examining the association between depression in the elderly and brain connectivity, the potential role of four neural networks [16–18, 23, 24] emerges in the pathophysiology of the disorder: the (1) Affective/Frontolimbic Network (anterior cingulate cortex (ACC), left and right amygdala and nucleus accumbens, and the left and right Orbitofrontal cortices (OFC)), the (2) Default Mode Network (Posterior Cingulate Cortex (PCC), Medial Prefrontal Cortex (MPFC), left and right Angular Gyri), the (3) Salience Network or ventral attention network (Anterior Cingulate Cortex, left and right Dorsolateral Prefrontal Cortex (DLPFC), left and right Anterior Insula, PCC), and the (4) Cognitive Control Network (Anterior Cingulate Cortex, left and right Dorsolateral Prefrontal Cortex (DLPFC), left and right posterior parietal cortex). We used the PCC as a seed for the connectivity calculations in the first two networks and the ACC in the latter. Based on a recent meta-analysis, we expected hypoconnectivity in these networks in patients with depressive symptoms [25]. There seems to be a considerable overlap between the functional networks studied in depression and cognitive decline. According to the meta-analysis of Eyler et al. [26], several studies found DMN impairments in MCI and AD. While the DMN is the most studied functional network in MCI, there are many inconsistencies across the findings. Another review by Teipel et al. [27] found a reduced correlation of resting state BOLD activity in the DMN and the attentional networks in MCI and AD. While in the case of depressive symptomatology, network malfunctioning seems to play a major role, in MCI, atrophy seems more critical. Therefore, we also performed conventional structural analyses on temporal and frontal lobe structures (e.g., the hippocampus or the orbitofrontal cortex), the precuneus, and white matter hyperintensities (WMH) to co-investigate the possible effects of MCI and depressive symptomatology. White matter hyperintensities (WMHs) are regions exhibiting heightened signal intensity, notably visible on T2-weighted MRIs. Several previous studies have identified a heightened frequency and increased severity of WMHs in elderly subjects with depressive symptoms [28] and subjects with vascular dementia [29].

### Graph theoretical analysis

While several previous research on LLD studies examined connectivity in the networks mentioned above, only a few studies examined whole-brain functional connectivity using graph theoretical techniques [30–32]. Researchers use graph-theoretical analyses to investigate the general patterns of whole-brain functional connectivity, which refers to communication patterns between distant brain regions. Among these approaches, the Minimum Spanning Tree (MST) method has gained popularity due to its robustness and ability to provide an impartial network representation and to overcome the thresholding problem [33, 34]. Therefore, MST is particularly suitable for comparing networks derived from different groups of subjects or networks with varying densities. Earlier research highlighted the dependence of graph theoretical measures on network size and density. This dependence poses challenges when comparing different groups and conditions using conventional analytical methods for networks [35–37]. By employing the MST calculation, these biases related to network density and degree are overcome. The MST creates an acyclic subnetwork by including the strongest connections without loops and ensuring all nodes are connected with a fixed number of edges ([number of nodes] - 1). MST-based analyses were successfully applied in previous studies of depression and cognitive decline [39, 40]. We intended to examine whole-brain functional connectivity by global network metrics as we analyzed (A) functional integration and segregation by Mean Eccentricity (~Average path length), and Network Diameter and (B) centrality by Maximum Betweenness Centrality and leaf fraction and (C) network resilience by degree divergence [38].

**Fig. 1:**
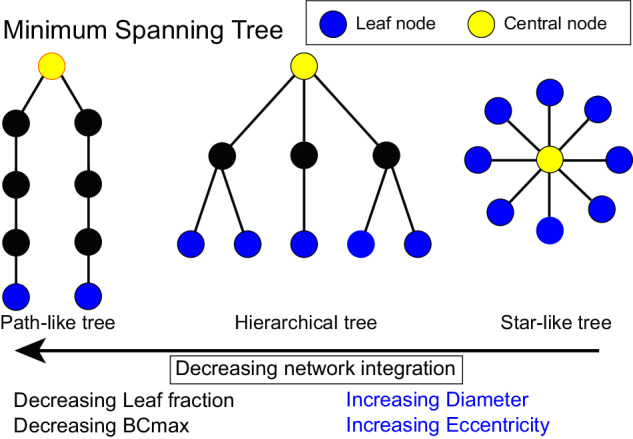
Illustrated are schematic depictions of three different types of minimum spanning trees (MSTs). These MST structures can vary, ranging from a tree resembling a linear path (indicating minimal integration within the network) to a star-like shape (indicating maximal integration within the network). In these representations, nodes in blue signify leaf nodes, essentially the endpoints of the graph, while nodes in yellow represent central nodes. The hierarchical tree design combines a relatively small diameter with a comparatively low betweenness centrality (BCmax) value. This combination prevents excessive information congestion at the central node, making it an ideal configuration for efficient network operation. [75]. The Figure was adjusted from van Dellen et al. [76], van Lutterveld et al. [77], and Fodor et al. [43].

Two extreme topologies of MST can be distinguished: a path-like (or line-like) and a star-like shape. In a path-like topology, all nodes are linked to exactly two other nodes, except the two nodes at the extremities of the tree. These nodes are connected to only one other node and are referred to as the leaves of the tree. This type of network is characterized by low centrality and integration. In the case of a star shape, all but one node is linked to a central node [37]. This other extremity is characterized by low network segregation and resilience. Between these two shapes, MST-s can have various configurations (Fig. 1), such as the structural and functional network topology of the healthy human brain, which can be characterized by high network integration and segregation together with good resilience. Therefore, the healthy brain demonstrates an optimal information processing system through a well-structured functional network with modular, hierarchical, balanced, and cost-efficient organization. This network, known as a small-world topology with rich clubs, ensures effective communication. However, various neurological and psychiatric conditions are associated with specific disruptions in connectivity and biased network structures [18, 37, 41, 42]. For instance, in conditions like depression or dementia, there is evidence of a compromised balance between segregation and integration within the brain’s functional network. Degree divergence measures the broadness of the degree distribution, which shows high value in networks with high-degree hubs and is related to the network’s resilience against attacks.

### Hypotheses

Based on previous studies, neurodegeneration and hub overload seem to change the network toward a highly centralized, more star-like topology with increased integration at the cost of decreased segregation and resilience [37, 43]. Therefore, impaired network centrality was expected in patients with MCI regarding decreased betweenness centrality and increased leaf fraction. On the other hand, depression changes the network topology into a more path-like form with reduced network integration [32]. Therefore, we expected an impaired network integration in the whole brain functional network in terms of increased Eccentricity and Diameter in subjects with depressive symptoms compared to non-depressed individuals. Finally, an impaired network resilience was expected in patients with depression and MCI subjects regarding decreased degree divergence. We wanted to explore the possible combined effect of MCI and depression on network topology. In other words, we studied if there is a different effect of depression on functional network configuration between subjects with MCI and healthy controls.

Regarding structural measures, we hypothesized that a higher degree of neural atrophy and WMH burden would be found in depressed and MCI subjects compared to non-depressed and cognitively unimpaired participants.

## Methods

### Ethics statement

The experiments were conducted in full compliance with the Helsinki Declaration and all relevant national and international ethical guidelines. The National Ethics Committee, Budapest, Hungary, approved the research. All procedures were carried out only after written informed consent was obtained from the participants. All potential participants who declined to participate or otherwise did not participate were not disadvantaged in any way by not participating in the study.

### Cohorts, subjects, and procedures

Data were gathered from 183 subjects in two separate research centers: (1) the Semmelweis MCI Neuroimaging Cohort (SMNC) and (2) the AlzEpi Cohort Observational Library (ACOL). The Euro-Fingers Consortium [44] facilitated the harmonization of the data. Participants were recruited from the Department of Psychiatry and Psychotherapy, Semmelweis University (SMNC database), and the National Institute of Mental Health, Neurology, and Neurosurgery (ACOL database). All participants were Hungarian natives. For further demographics, see Table 1. This was an exploratory study. Empirical and feasibility considerations determined the number of enrolled subjects. No formal statistical sample size estimation was performed.

**Table 1:** Demographics and Neuropsychology.

	HC non-DEP (*n* = 53)	HC DEP (*n* = 18)	naMCI non-DEP (*n* = 47)	naMCI DEP (*n* = 17)	aMCI non-DEP (*n* = 36)	aMCI DEP (*n* = 12)
Mean Age (SD)	67.5 (7.1)	64.4 (5.8)	71.7 (6.1)	71.4 (7.8)	70.7 (7.1)	74.5 (8.6)
Education (high)^a^	51.0%	72.2%	48.9%	58.8%	52.8%	16.7%
Gender (Female)	64.1%	83.3%	66.0%	82.3%	44.4%	41.7%
Rey Auditory Verbal Learning Test 1-5 sum^b^	51.3 (8.1)	51.4 (8.8)	46.4 (8.4)	44.0 (10.1)	30.8 (8.8)	22.6 (7.7)
Rey Auditory Verbal Learning Test delayed recall^c^	10.9 (2.5)	10.4 (2.6)	9.7 (3.2)	8.9 (2.9)	4.0 (2.8)	3.5 (2.6)
ACE Total Score^d^	94.2 (3.3)	93.4 (3.0)	89.6 (6.3)	87.2 (5.2)	85.1 (8.9)	79.0 (8.1)
ACE VL/OM-ratio^e^	2.5 (0.3)	2.5 (0.4)	2.6 (0.5)	2.6 (0.4)	3.0 (0.7)	3.1 (1.0)
MMSE Total Score^f^	28.5 (1.2)	28.6 (1.2)	28.1 (1.4)	28.1 (1.4)	27.8 (1.4)	27.3 (1.4)
Trail Making Test Part A^g^	40.0 (12.1)	45.6 (15.9)	52.3 (21.8)	71.1 (47.0)	67.1 (51.3)	99.2 (124.1)
Trail Making Test Part B^g^	75.8 (22.7)	78.1 (22.2)	136.9 (55.6)	190.1 (118.8)	161.5 (90.1)	204.2 (119.6)
Depression z-score	−0.56 (0.52)	1.39 (0.79)	−0.39 (0.52)	1.38 (0.74)	−0.44 (0.51)	1.28 (0.66)

Standard Deviations (SD) in brackets.

*HC* healthy control, *naMCI* non-amnestic Mild cognitive impairment, *aMCI* amnestic Mild cognitive impairment, *DEP* subjects showing depressive symptoms, *non-DEP* subjects not showing depressive symptoms, *ACE* Addenbrooke’s Cognitive Examination, *MMSE* Mini-Mental State Examination.

^a^Participants were categorized into three education groups: 1=less than 12 years; 2=high school graduation (12 years education); 3=more than 12 years of education.

^b^Sum of all words in the first five trials. The maximum score is 75.

^c^The maximum score is 15.

^d^The maximum score is 100.

^e^VL/OM: verbal fluency and language points/orientation and delayed recall ratio can be defined based on ACE. A score below 2.2 indicates frontotemporal dementia, while a score over 3.2 indicates Alzheimer’s disease.

^f^The maximum score is 30.

^g^Time needed for completing the task in seconds.

Inclusion criteria were (1) the age of >= 55 years, (2/A) diagnosis of MCI according to the Petersen criteria (see below), (2/B) no cognitive deficit present (healthy control group). Exclusion criteria were (1) the history of unconsciousness for more than an hour (2) CNS infectious disease, (3) clinically significant brain lesions (stroke, severe periventricular white matter disease, clinically significant white matter infarcts), (4) alcohol or other substance use or dependency, (5) mental retardation (6) multiple sclerosis or other demyelinating disorders, (7) hydrocephalus, (8) untreated vitamin B12 deficiency, (9) untreated hypothyroidism, (10) syphilis or HIV infection (11) major neurocognitive disorder defined as a <= 24 score on the MMSE.

Participants underwent a comprehensive evaluation of their neurological and neuropsychological condition conducted by neuropsychologists, neurologists, or trained neuroscientists. Blood tests, cerebrospinal fluid (CSF) analysis (in a small subgroup of subjects), and MRI scans were also performed. The neuropsychological assessment battery included the Hungarian version of the Rey Auditory Verbal Learning Test, the Hungarian version of the Addenbrooke’s Cognitive Examination (including the Mini-Mental State Examination: MMSE), and the Trail-making Test A and B. A total of six study groups were formed based on two main criteria, neurocognitive status and the presence of above-threshold depressive symptoms.

Regarding cognitive status, we enrolled participants categorized into three groups: those with amnestic MCI (aMCI), those with non-amnestic MCI (naMCI), and healthy controls (HC), all according to the Petersen criteria [45]. These criteria involve the presence of subjective memory complaints supported by an informant, the maintenance of everyday activities, evidence of memory impairment through a standard neuropsychological test, intact overall cognitive functions, and the exclusion of dementia. However, the Petersen criteria do not specify a neuropsychological test for assessing memory impairment. Therefore, we used the Rey Auditory Verbal Learning Test (RAVLT), the most commonly employed test in the literature [46].

To differentiate between individuals with aMCI and healthy controls, we employed a cutoff score of 1 standard deviation below the population mean, which was standardized for age and gender. Those who fell below this cutoff value either in the delayed recall subscore or the total score were categorized as having aMCI. These criteria align with the recommendations of the National Institute on Aging - Alzheimer’s Association workgroups on diagnostic guidelines for Alzheimer’s disease [47].

For individuals not falling into the aMCI group but scoring one standard deviation below the population mean (standardized for age, gender, and education) either in the Trail Making Test B or the Addenbrooke’s Cognitive Examination (ACE), they were categorized into the naMCI group. An additional criterion for the naMCI group was a VLOM (verbal fluency + language score/orientation + memory score) ratio lower than 3.2 in the ACE to exclude potential aMCI cases from the naMCI group (these participants were excluded from the study.).

To evaluate the potential presence and severity of depression, the 13-item version of the Beck Depression Inventory (BDI) [48] or the Geriatric Depression Scale (short form) (GDS) was utilized [49, 50]. The potential presence of depression (i.e., caseness) was defined as a score of >= 10 on the BDI and >= 5 on the GDS. We will refer to subjects above these cutoff scores as a ‘depressed subgroup’ (aMCI depressed, naMCI depressed, healthy control depressed) in the manuscript. However, it is important to note that a depression questionnaire score does not necessarily indicate the presence of major depression. Its diagnosis requires a specialist psychiatric examination or a structured clinical interview. Therefore, a proportion of subjects described in this manuscript as ‘depressed subgroups’ are most likely to have suffered from subclinical depression. In order to make depression assessment comparable in correlational analyses, z scores were calculated from both depression measures as per the following formula: z score = (*x*−µ)/σ (*x* = individual measurement, µ = subgroup mean, σ = subgroup standard deviation).

### MRI examinations

Participants underwent brain 3 Tesla MRI using three protocols since the SMNC included two cohorts. All three imaging procedures were allowed for acquiring high-resolution anatomical images and functional MRI data, enabling further analysis and investigations in the study. All protocols consisted of a T2-, diffusion-, and a FLAIR-weighted sequence to identify the possible pathological lesions. During the “resting-state” functional MRI acquisition, participants were instructed to fixate on a cross displayed at the center of the screen. Participants were explicitly informed to report if they fell asleep during the recording, and none of the subjects reported doing so. Foam padding was used to minimize head motion artifacts. For further details on the scanners and imaging protocols, see Supplement Table 1. See the *supplement* for a detailed description of MRI preprocessing by the CONN toolbox and MRI structural analysis by Freesurfer.

### Functional MRI connectivity analysis

The CONN toolbox provides a series of default pre-defined regions we used for connectivity analyses. These ROIs include a complete brain parcellation of 91 cortical areas and 15 subcortical regions from the FSL Harvard-Oxford Atlas [51–54], as well as 26 cerebellar areas from the AAL atlas [55] and a series of hub regions characterizing the Default Mode Network (DMN), the dorsal attention network, and the executive control network.

We calculated the ROI-to-ROI connectivity matrices representing the functional connectivity between each pair of ROIs [56]. Each element in the connectivity matrix is defined as the Fisher-transformed bivariate correlation coefficient between a pair of ROI BOLD time series. For the exact mathematical formula, see https://web.conn-toolbox.org/fmri-methods/connectivity-measures/roi-to-roi.

### Graph-theoretical analysis

The functional connectivity matrix was transformed into a graph-theoretical representation using the Minimum Spanning Tree (MST) method. This approach creates a simplified core network model, capturing the strongest and most relevant connections. The MST graph reflects topological changes and has been previously used in studies [37, 57]. For each participant, MST graphs were generated based on the full connectivity matrix derived from the connectivity values obtained for each pair of ROIs by the CONN toolbox.

The tree’s *diameter* is the maximum number of edges between any two nodes of the network. *Leaf fraction* is the number of nodes with exactly one connection divided by the total number of nodes of the tree. *Degree* refers to the number of edges connected to a node. The *betweenness centrality (BC)* of a node refers to the normalized fraction of all paths connecting two nodes that pass through the selected node, and it characterizes the ‘hubness’ of the node within the network. The *eccentricity* of a node denotes the maximum distance to any other node in the MST. *Degree divergence* (kappa - κ) measures the broadness of the degree distribution, which shows high value in networks with high-degree hubs and is related to the network’s resilience against attacks. The most efficient communication in an MST can be achieved in a star-like configuration, as it has the shortest possible *average path length* (~Mean Eccentricity) between two arbitrary nodes. However, in this case, the central node might easily be overloaded.

Global and node-specific parameters were computed in MATLAB based on the measures described by previous studies [37, 38, 57]. Degree, betweenness centrality (BC), and eccentricity were calculated for each node separately, and the degree divergence, maximum BC, and mean eccentricity were included in the statistical analysis as global characteristics of the MST.

### Structural MRI analysis

We examined the cortical thickness and the subcortical structures’ volume in a selection of structures. The choice was based on previous results [58] on differentiating MCI from healthy aging and papers summarizing possible structural differences in LLD [17]. Furthermore, the cortical thickness and volume of the major hubs of the functional network were also analyzed. Altogether the following ten structures were selected for either volume analysis (1) the amygdale, (2) the hippocampus, (3) the accumbens area or cortical thickness calculations (4) the precuneus, (5) the entorhinal cortex, (6) the isthmus of the cingulate gyrus, (7) the parahippocampal gyrus, (8) the orbitofrontal cortex, (9) the anterior cingulate gyrus, and (10) the fusiform gyrus. The total volume of (11) white matter hyperintensities (WMH burden) was also analyzed. White matter hyperintensities were assessed by measuring white matter hypointensities in Freesurfer on T1-weighted images. Based on previous research by Wei et al. [59] WMH on T2-weighted (T2 FLAIR) images and white matter hypointensities on T1-weighted images are highly correlated (*r* > 0.8).

### Statistical analyses, including sensitivity analyses

A General Linear Model analysis (PROC GLM in SAS) was conducted to examine the effect of depression and MCI and their interaction on connection strengths in the FLN, DMN, SN, and CCN. The interaction of depression and MCI was included in all GLM analyses in order to study the possible different effects of depressive symptoms on network functionality in MCI and cognitively healthy subjects. Furthermore, the analyses included MRI scanner type [60], sex, age, education, and total connectivity strength as covariates. The same model was applied to analyze differences in network metrics and structural measures (e.g., cortical thickness and volumes). We included the total connection strength (i.e., a mathematical sum of all connection strengths) as a covariate in the connection strength analyses suggested by van den Heuvel et al. [33] to overcome the bias introduced by the significant between-subject variance in connection strengths in network analyses. All p values were corrected for multiple comparisons by the Bonferroni method as follows: (1) corrected *p* = 0.05 / (number of hubs-1) in the network (number of hubs in FLN = 7, DMN = 4, SN = 8, CCN = 5) or (2) corrected *p* = 0.05/network parameters (*p* = 0.05/5 = 0.01).

Twenty-two subjects took antidepressant (AD) treatment in the whole sample (*n* = 12 in the depression subgroup and *n* = 10 in the non-depressed subgroup). To prove that these medications do not affect our main functional results, we performed all primary analyses with and without these subjects as a sensitivity analysis.

Since the sample size was low in one of the MCI subgroups (aMCI/Dep, *n* = 12), we merged the MCI subgroups and repeated the primary analyses with MCI as a binary variable (0 = HC; 1 = MCI).

## Results

### Demographics and depressive symptoms

Age and education level did not show correlations with depressive symptom severity (*p* > 0.1). There was a statistical trend of more female than male subjects with depression; however, this difference did not reach significance (29.6% vs. 19.1%; Chi-Square = 2.4, *p* = 0.12).

### Correlation between cognitive functioning and depression symptom severity

Depressive symptoms severity in terms of z scores showed negative correlations with ACE total score and positive correlation with Trail Making A and B times (Table 2). Correlations of depressive symptoms with RAVLT and MMSE were non-significant (*p* > 0.1). The frequency of caseness regarding depressive symptoms did not differ between the HC (25.3%) and MCI (aMCI and naMCI) (26.1%) groups (Chi-Square = 0.01, *p* = 0.91).

**Table 2:** Correlation between cognitive functioning and depression symptom severity.

	Pearson *r*	*p*-value	Spearman *r*	*p*-value
ACE Total score	−0.20*	0.006	−0.23*	0.002
Trail Making A time	0.18*	0.02	0.23*	0.002
Trail Making B time	0.17*	0.02	0.16*	0.04
Rey Verbal Learning Test	−0.11	0.13	−0.14	0.07
Mini-Mental State Examination	−0.08	0.26	−0.12	0.11

Asterisk indicate significant correlation (*p* < 0.05).

### Network parameters

**Fig. 2:**
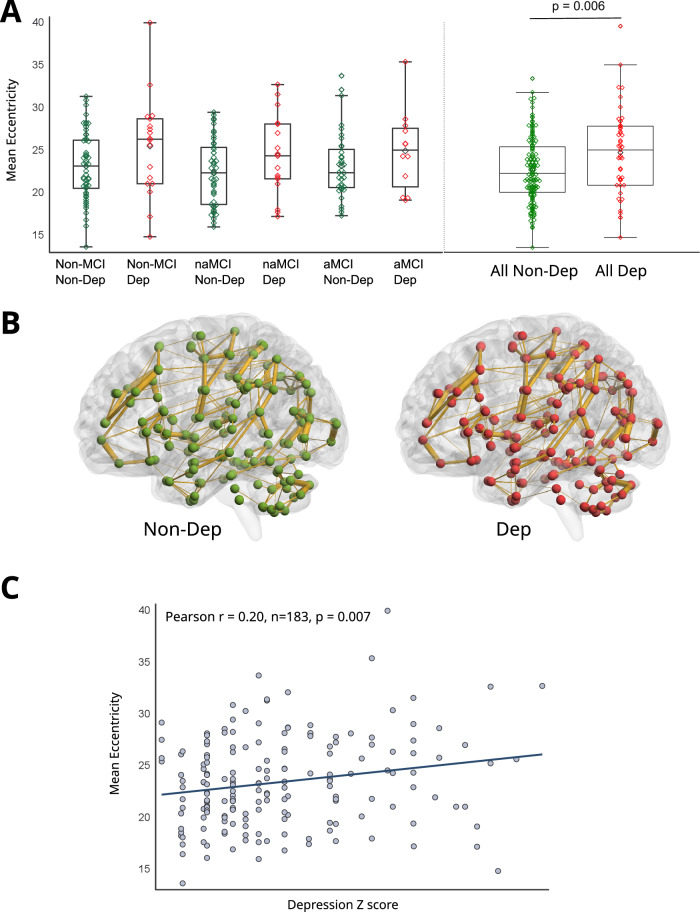
Mean Eccentricity in the Minimum Spanning Tree network and depressive symptoms. **A** Mean Eccentricity in the study groups. non-DEP: subjects without depressive symptoms; DEP: subjects showing depressive symptoms; aMCI = amnestic Mild Cognitive Impairment; naMCI: non-amnestic Mild Cognitive Impairment. The display includes a box spanning the Q1-Q3 inter-quartile range, with a line drawn at the median value. A black diamond marks the mean value. **B** A grand average version of the Minimum Spanning Tree networks in subjects with (DEP) and without depressive symptoms (Non-Dep). Connections present in at least 10% of the subjects are drawn for clarity [78]. **C** Correlation between Mean Eccentricity and depressive symptom severity in terms of z-scores (all subjects).

An investigation was carried out using the General Linear Model analysis to explore how depression and MCI and their interaction influence the network measures. The analyses included covariates such as MRI scanner, sex, age, education, and total connectivity strength. We assessed functional integration by Mean Eccentricity (~Average Path Length) and Diameter in the MST. We found an increased Mean Eccentricity (Fig. 2A; *F*(1182) = 7.9, *p* = 0.006; LS-Means (SE): CNTRL = 23.2 (0.4), DEP = 25.2 (0.6)) and Diameter (*F*(1182) = 6.9, *p* = 0.009; LS-Means (SE): CNTRL = 30.3 (0.5), DEP = 32.8 (0.8)) in patients showing depressive symptoms. MCI and its interaction with depression had no significant effect (*p* > 0.1). Repeating the analysis excluding subjects on AD treatment did not change the results. We also repeated the analysis, including MCI as a binary variable, by merging the two MCI subtypes (Supplement Fig. 1; *F*(1182) = 8.5, *p* = 0.004) and Diameter (*F*(1182) = 7.5, *p* = 0.007).

Mean Eccentricity (Fig. 2C; Pearson *r* = 0.20, *n* = 183, *p* = 0.007; Spearman *r* = 0.19, *n* = 183, *p* = 0.009) and Diameter (Pearson *r* = 0.18, *n* = 183, *p* = 0.01; Spearman *r* = 0.17, *n* = 183, *p* = 0.02) showed a positive correlation with depression severity (in terms of z scores) as measured on the GDI or BDI. Repeating this correlational analysis excluding subjects on AD treatment did not change the results.

Patients with depressive symptoms showed decreased network resilience in terms of degree divergence (*F*(1,182) = 3.9, *p* = 0.0498; LS-Means (SE): CNTRL = 1.12 (0.007), DEP = 1.09 (0.011)). However, this effect did not reach significance after correction for multiple comparisons. MCI and its interaction with DEP had no significant impact (*p* > 0.1). There was a statistical trend-level negative correlation between depressive symptom severity and degree divergence (Pearson *r* = − 0.14, *n* = 183, *p* = 0.07; Spearman *r* = − 0.14, *n* = 183, *p* = 0.07).

Centrality in terms of betweenness centrality and leaf fraction did not differ between groups (DEP vs. non-DEP or HC vs. MCI), nor did it show a correlation with depressive symptom severity (all *p* values > 0.1).

All analyzed network parameters in the study groups are presented in Supplement Table 2.

### Functional connectivity in the affective/frontolimbic network, the default mode network, the salience network, and the cognitive control network

A General Linear Model analysis was conducted to examine the effect of depression and MCI and their interaction on connection strengths in the DMN, SN, CCN, and FLN. The analyses included covariates such as MRI scanner, sex, age, education, and total connectivity strength.

**Fig. 3:**
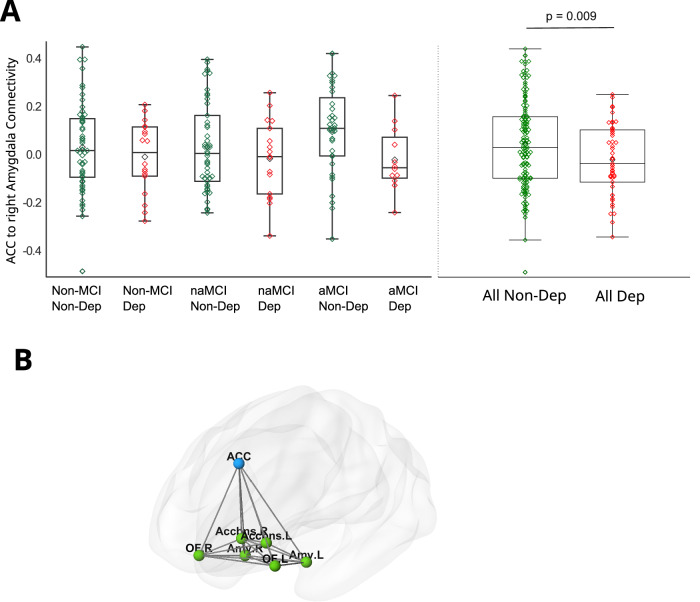
Functional connectivity in the Frontolimbic network. **A** Anterior Cingulate Cortex (ACC) to right Amygdala functional connectivity in the Forntolimbic network. DEP: subjects showing depressive symptoms; non-DEP: subjects without depressive symptoms; aMCI = amnestic Mild Cognitive Impairment; naMCI: non-amnestic Mild Cognitive Impairment; Non-MCI: subjects without MCI. The display includes a box spanning the Q1–Q3 inter-quartile range, with a line drawn at the median value. A black diamond marks the mean value. **B** A schematic image depicting the Frontolimbic network consisting of seven hubs: the Anterior Cingulate Cortex, the left and right Orbitofrontal Cortex, the left and right Amygdale, and the left and right nuclei Accumbens.

In the frontolimbic network, the ACC to right Amygdala (Fig. 3; *F*(1,182) = 8.9, *p* = 0.003; LS-Means (SE): CNTRL = 0.07 (0.02), DEP = −0.02 (0.03)) connectivity was weaker in patients showing depressive symptoms. We repeated the analysis, including MCI as a binary variable, by merging the two MCI subtypes (Supplement Fig. 2; *F*(1,182) = 7.9, *p* = 0.005; LS-Means (SE): CNTRL = 0.06 (0.02), DEP = −0.03 (0.03)). This connection strength also significantly correlated with depressive symptom severity as z-scores (Pearson *r* = −0.17, *n* = 183, *p* = 0.02; Spearman *r* = −0.15, *n* = 183, *p* = 0.049), while it did not correlate with the volume of the Amygdale or the thickness of the anterior cingulate gyrus (all p values > 0.1). Repeating these two analyses without subjects on AD treatment did not change the results. The ACC to left Amygdala connection was stronger in patients with MCI than the cognitively unimpaired controls (*F*(1,182) = 3.5, *p* = 0.03; LS-Means (SE): CNTRL = −0.03 (0.03), MCI = 0.07 (0.03)). However, this latter did not reach significance after controlling for multiple comparisons.

In the DMN, the connectivity between the PCC as the central hub (seed) and the left Angular Gyrus was decreased in patients showing depressive symptoms (*F*(1,182) = 5.1, *p* = 0.02; LS-Means (SE): CNTRL = 0.65(0.02), DEP = 0.55(0.04)). However, this latter did not reach significance after correction for multiple comparisons. The interaction of DEP and MCI was non-significant in all cases. In the SN and CCN, the effect of DEP, MCI, and their interaction on connection strengths did not reach significance (*p* > 0.1).

### Network hubs in the minimum spanning tree

In a descriptive analysis, we averaged the MSTs in depressed and non-depressed subjects (Fig. 2B). We ranked all 164 CNS structures according to their number of connections (edges) to other nodes. The two primary hubs in the studied networks, the ACC and PCC, were in the upper 25% percentile in both study groups: the PCC was 17th (upper 10% percentile), and the ACC was 35th (upper 25 percentile) in the non-depressed subjects, while the PCC was 31st (upper 25 percentile), and the ACC was 39th (upper 25 percentile) in depressed subjects. Among the other nodes in the analyzed networks, the MPFC, the left and right anterior insula, and the angular gyri were in the upper 10% percentile in both groups. The left and right DLPFCs were in the upper 50% percentile, while the frontal orbital cortices, the amygdale, and the nuclei accumbens were in the lower 50% percentile in both groups.

### Structural MRI analyses

**Fig. 4:**
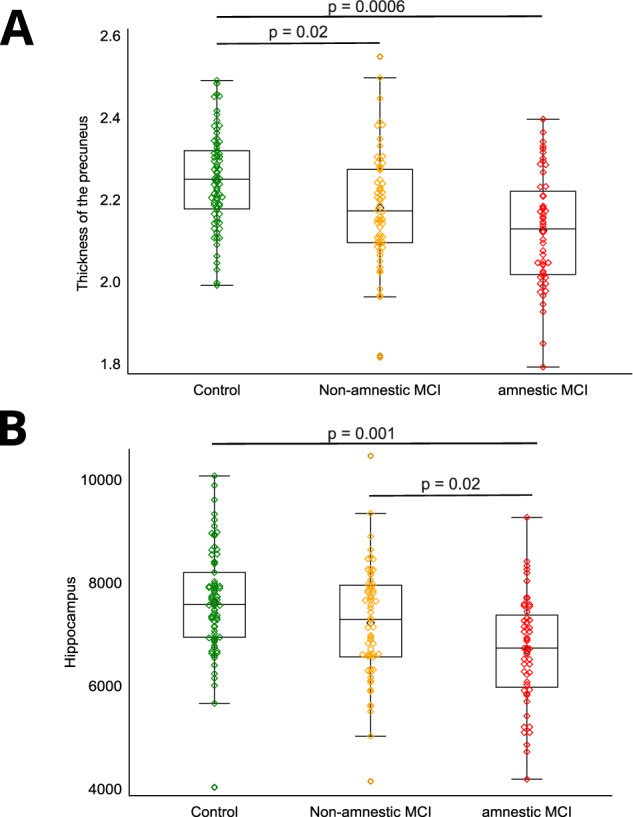
Structural differences between controls and MCI subgroups. **A** Thickness of the precuneus (mm) aMCI = amnestic Mild Cognitive Impairment; naMCI: non-amnestic Mild Cognitive Impairment; Control: subjects without MCI. The display includes a box spanning the Q1-Q3 inter-quartile range, with a line drawn at the median value. A black diamond marks the mean value. **B** Volume of the hippocampus (mm3) aMCI = amnestic Mild Cognitive Impairment; naMCI: non-amnestic Mild Cognitive Impairment; Control: subjects without MCI. The display includes a box spanning the Q1-Q3 inter-quartile range, with a line drawn at the median value. A black diamond marks the mean value.

There was a significant difference between patients with MCI and controls in the thickness of the precuneus (*F*(2, 177) = 7.7, *p* = 0.0006; post-hoc test: Control>naMCI (*p* = 0.018), Control > aMCI (*p* = 0.0006), naMCI=MCI (*p* = 0.35) (Fig. 4)) and in the volume of the Hippocampus (*F*(2, 177) = 6.6, *p* = 0.0018; post-hoc test: Control = naMCI (*p* = 0.55), Control > aMCI (*p* = 0.001), naMCI > MCI (*p* = 0.019) (Fig. 4)), while the thickness of the entorhinal cortex showed a statistical trend level difference (*F*(2, 177) = 5.6, *p* = 0.0046; post-hoc test: Control = naMCI (*p* = 0.82), Control > aMCI (*p* = 0.004), naMCI > MCI (*p* = 0.03)). There was no statistical difference in any structure’s thickness or volume, including WMH, between subjects showing depressive symptoms and non-depressed subjects (*p* > 0.05). Also, the interaction of MCI and depression did not significantly affect volumes or cortical thickness (*p* > 0.05). All analyzed structural measures in the study groups are presented in Supplement Table 2.

The possible associations between CNS structures and network parameters were analyzed by Pearson correlations. None of the above structures correlated significantly with mean eccentricity, diameter, leaf fraction, or betweenness centrality (*p* > 0.05).

## Discussion

To our knowledge, this is the first study investigating the combined effects of mild cognitive impairment and depressive symptomatology on functional brain network topology by MST on a large sample of elderly subjects. We found an impaired whole-brain network integration and decreased functional connectivity in the Frontolimbic Network in elderly patients showing depressive symptoms.

An increased mean eccentricity and network diameter were found in patients with depressive symptoms, and both network measures showed correlations with depressive symptom severity. The increase in eccentricity and diameter indicates a more path-like functional network configuration. Path-like network topology may lead to an impaired functional integration in depression, which may be the underlying cause of depressive symptomatology in the elderly. Previous studies found similar results regarding global connectivity [30–32]. The interaction effect of impaired cognition (MCI groups) and depression on network parameters was non-significant, and no comparable difference in global network measures was found between MCI and cognitively unimpaired subjects. Furthermore, functional integration did not correlate with cognitive performance. Also, the difference in network measures between depressed and non-depressed subjects was found in cognitively unimpaired and MCI subjects. Therefore, this finding seems to be depression specific and independent of cognition in the elderly. The lack of correlation of structural measures with functional connectivity and graph theoretical parameters (e.g., mean eccentricity) also supports this notion. Previous EEG studies [43] showed an increased centralization in terms of increasing betweenness centrality and a more star-like configuration, indicating a more centralized topology in cognitively impaired subjects, which is an opposite process compared to what we found in the case of depression. No similar difference between MCI and cognitively unimpaired subjects was found in the present study. A possible explanation is that subjects in the current investigation were only slightly impaired cognitively and might not show these network impairments. Another possible explanation is that EEG connectivity is a more sensitive measure of early functional network impairments in MCI than fMRI. Degree divergence, a measure of network resilience against attacks, was decreased in depressed subjects and correlated with depressive symptom severity at a trend level. This result is in line with previous studies showing lower resilience against failure in the brain networks of depressed subjects [31].

A functional hypoconnectivity was detected in patients with depressive symptoms between the ACC and the right amygdala in the affective/frontolimbic network, which impairment correlated with depressive symptom severity. The affective/frontolimbic network comprises interconnected neural structures, including the amygdala, the ACC, the OFC, and the nucleus accumbens. This network primarily serves two functions: emotional processing and mediation of motivated behaviors [18]. Additionally, it plays a crucial role in regulating the connection between emotions and moods with visceral functions. Various studies have demonstrated significant involvement of dysfunction in the affective/frontolimbic network in mood and depressive disorders [61, 62]. We also found a tendency-level hypoconnectivity in the DMN between the PCC and the left Angular gyrus, which aligns with many previous studies finding impairments in the DMN [16]. Other studies found hypoconnectivity in the CCN and SN [18, 63, 64], while we found no impairments in patients with depressive symptoms in these networks. A possible explanation for this discrepancy is that the studies above examined patients with LLD, while the present investigation mainly included subjects with subclinical depression. We would like to note that there is no clear consensus on what functional brain networks are impaired in LLD; however, most studies find hypoconnectivity in the Frontolimbic and Default Mode networks. We did not find proof that depressive symptoms affect patients with MCI differently than cognitively unimpaired subjects. We ranked the studied network hubs in the MST according to their connection number. We found that the two major hubs, the PCC and the ACC, were in the upper 25% percentile, indicating that these are essential central hubs critical for general information transmission and circuit-level computing.

Due to the low sample size in the ‘aMCI / DEP’ subgroup, we conducted sensitivity analyses by merging the MCI subgroups and repeating the primary analyses to test the reliability of the results. The findings did not change, proving that the results are not a result of the subgrouping.

Subjects with depressive symptoms performed worse on the ACE and Trail Making A and B tests, while there was no difference regarding depression frequency (i.e., caseness; see criteria in the methods section) between healthy and MCI subjects. The correlation of depression symptom scores with cognitive measures aligns with previous studies [65, 66] and might indicate a general cognitive impairment in patients with depression. This finding, taken together with data from the literature and prior results showing no association between Amyloid deposition and depressive symptoms [23], rather indicates a higher risk for cognitive decline in depression than being an early sign of dementia [67, 68].

While no structural difference was found between subjects with depressive symptoms and non-depressed controls, the volume of the hippocampus and the thickness of the precuneus and the entorhinal cortex differed between subjects with MCI and cognitively healthy controls. The latter two also differed between the two subtypes of MCI, showing more severe atrophy in the case of aMCI than naMCI. This finding aligns with the literature and our previous results [58, 69, 70] as aMCI is considered the risk group for Alzheimer’s disease. Some previous investigations found structural impairments in various brain regions in LLD [17]; however, amyloid burden and the subclinical depressive symptoms were not associated [23], suggesting different pathophysiological mechanisms for cognitive and depressive symptoms in AD. A possible reason that the present study did not find any similar impairments is that we applied stringent inclusion criteria regarding cognitive impairment to exclude subjects with dementia.

### Limitations

The multi-center study was designed to detect and monitor cognitive impairments in the elderly and not to follow up on late-life depression. Therefore, structured clinical interviews for psychiatric disorders such as the SCID or MINI were not assessed. Furthermore, different measures of depression (GDS and 13-item BDI) were applied in the two study centers. However, the dimensional conceptualization of psychiatric disorders has emerged as cognitive and affective neuroscience revealed neural systems using neuroimaging techniques such as functional MRI. Therefore, analysis from a dimensional perspective aligns with a state-of-the-art view, such as the Hierarchical Taxonomy of Psychopathology [71] and Research Domain Criteria [72]. Also, it is motivated by the general psychometric and ethical arguments favoring dimensional over categorical indicators [73]. Therefore, analysis from a dimensional perspective aligns with a state-of-the-art view of psychiatric disorders. Alzheimer’s biomarkers, such as Beta-Amyloid and Tau, as suggested by the international guidelines [74], were not measured. In the present study, only functional connectivity was analyzed. No indices of structural connectivity, such as fractional anisotropy or mean diffusivity, were included. Study groups slightly differed in demographical parameters, such as educational level, age, and gender; therefore, all these variables were included in the analyses as covariates. Some patients (*n* = 22) were on AD treatment; however, all alterations remained significant when we excluded these subjects from the analyses.

## Conclusion

In line with previous results, structural impairments such as cortical thinning of the precuneus and hippocampal volume loss can be associated primarily with early cognitive decline. At the same time, depressive symptoms are connected to functional network properties such as mean hub eccentricity, network diameter, or degree divergence without severe structural brain atrophy. These network impairments result in decreased functional integration and network resilience, which seem independent of cognitive impairments.

### Supplementary information

Supplement

## Data Availability

The data that support the findings of this study are available from the corresponding author, [Gábor Csukly: csukly.gabor@semmelweis.hu; csugab@yahoo.com], upon reasonable request.
