# Supplementary material for: Low Functional network integrity in cognitively unimpaired and MCI subjects with depressive symptoms: results from a multi-center fMRI study
Source: Transl Psychiatry. 2024 Apr 5;14:179. doi: 10.1038/s41398-024-02891-2 (PMC10997664; doi:10.1038/s41398-024-02891-2)
Supplement: Supplementary file 1 — Supplement [file 41398_2024_2891_MOESM1_ESM.docx]

**Supplementary Information**

**Low Functional network integrity in cognitively unimpaired and MCI subjects with depressive symptoms: Results from a multi-center fMRI study**

MRI preprocessing

We used the CONN toolbox ^1^ to analyze resting-state fMRI data. The standard fMRI preprocessing pipeline consisted of the following steps: (1) Functional realignment and unwarp: The functional data were aligned to correct head motion artifacts. Additionally, unwarping techniques were applied to minimize distortions caused by magnetic field inhomogeneities. (2) Slice-time correction: If the data were acquired using Siemens' scanner (ascending) or Philips' scanner (interleaved), slice-time correction was performed to account for temporal differences between slices. (3) Outlier detection and scrubbing: Outliers in the functional data were identified using the Artifact Detection Toolbox (ART). These outlier scans were flagged for further scrubbing. (4) Direct functional and structural segmentation: Functional and structural data were segmented to separate gray matter, white matter, and cerebrospinal fluid (CSF). This segmentation process was performed simultaneously, and the resulting segmentation masks were used for subsequent analyses. (5) Normalization: The functional and structural data were normalized to the standard Montreal Neurological Institute (MNI) space. This normalization involved aligning the data to a standardized template, allowing for spatial comparability across participants. (6) Spatial smoothing: A spatial smoothing step was applied to the functional data to reduce noise and enhance the signal-to-noise ratio. The half-height width of the smooth kernel was 8mm. This process involved applying a Gaussian filter to the data.

Following the preprocessing procedures, an extra assessment was performed to evaluate the precision of the segmentation. Afterward, a band-pass filter was implemented, restricting the frequency range of the data to 0.008-0.09 Hz. This filter eliminated physiological artifacts and discarded irrelevant portions of the measured signal.

In the final stage, linear regression was utilized to eliminate undesired components from the data, which included removing signals originating from white matter and cerebrospinal fluid (CSF) and accounting for the effects of realignment and scrubbing. By employing this regression approach, potential sources of noise and confounding factors were further eliminated, thereby refining the data for subsequent analyses.

Structural MRI analysis

Cortical reconstruction and volumetric segmentation were conducted using the Freesurfer 7.3.2 image analysis suite, available for free download online at http://surfer.nmr.mgh.harvard.edu/. We adhered to the established procedures outlined in prior publications and did not modify this pipeline. The image processing workflow involved several steps in providing a brief overview: (1) Motion correction ^2^, (2) Removal of non-brain tissue through a hybrid watershed/surface deformation method ^3^, (3) Automated Talairach transformation, (4) Segmentation of subcortical white matter and deep gray matter volumetric structures, including the hippocampus, amygdala, caudate, putamen, and ventricles ^4^ (5) Intensity normalization (6) Tessellation of the gray matter-white matter boundary (7) Automated topology correction (8) Surface deformation guided by intensity gradients to accurately position the gray/white and gray/cerebrospinal fluid borders at the point where the most significant intensity shift indicates the transition to the other tissue class ^5^

Following the completion of cortical models, Freesurfer employed various deformable procedures for further data processing and analysis. These steps included (I) Surface inflation ^4^, (II) Registration to a spherical atlas that utilized individual cortical folding patterns to align cortical geometry across subjects ^6^, (III) Parcellation of the cerebral cortex into units based on gyral and sulcal structure ^7^ (IV) Creation of various surface-based data, such as curvature and sulcal depth maps.

Each subject's cortical models and segmentation results ultimately underwent quality checks and manual corrections. However, these corrections did not yield any significant alterations to the results.

| **Supplement Table 1** | | | |
| --- | --- | --- | --- |
|  | **ACOL** | **SMNC 1** | **SMNC 2** |
| scanner | Siemens Magnetom Verio 3T | 3 Tesla Philips Achieva | 3T Philips Ingenia MRI |
| manufacturer | Siemens Healthcare, Erlangen, Germany | Philips Medical Systems, Best, The Netherlands | Philips Medical Systems, Best, The Netherlands |
| head coil | 12-channel head coil | 8-channel head coil | 32-channel head coil |
| **Anatomical imaging** |  |  |  |
| sequence | T1-weighted 3D MPRAGE: magnetization prepared rapid gradient echo | T1W 3D Turbo Field Echo: T1-weighted three-dimensional spoiled gradient echo | T1W 3D Turbo Field Echo: T1-weighted three-dimensional spoiled gradient echo |
| repetition time (TR in ms) | 2.30 | 9.70 | 9.85 |
| echo time (TE in ms) | 3.40 | 4.60 | 4.60 |
| flip angle | 12° | 8° | 8° |
| field-of-view (FOV) |  | 240 mm × 240 mm | 240 mm × 240 mm |
| voxel size (mm) | 1.0 x 1.0 x 1.0 mm | 1.0 x 1.0 x 1.0 mm | 1.0 x 1.0 x 1.0 mm |
| **Resting-state functional imaging** |  |  |  |
| sequence | T2*-weighted echo-planar imaging (EPI) | T2*-weighted echo-planar imaging (EPI) | T2*-weighted echo-planar imaging (EPI) |
| duration (mins) | 10 | 8.5 | 8.5 |
| repetition time (TR in ms) | 2000 | 2000 | 1000 |
| echo time (TE in ms) | 30 | 30 | 25 |
| flip angle | 79° | 70° | 65° |
| field-of-view (FOV) |  | 240 mm × 240 mm | 240 mm × 240 mm |
| voxel size | 3.0 × 3.0 × 3.0 mm | 3.0 × 3.0 × 4.0 mm | 2.5 × 2.5 × 2.5 mm |

| **Supplement Table 2 Network Parameters and Structural Measures** | | | | | |  |
| --- | --- | --- | --- | --- | --- | --- |
|  | **HC non-DEP (n=53)** | **HC DEP (n=18)** | **naMCI non-DEP (n=47)** | **naMCI DEP (n=17)** | **aMCI non-DEP (n=36)** | **aMCI DEP (n=12)** |
| Network Parameters | | | | | | |
| ** Mean Eccentricity | 23.1 (4.0) | 25.4 (5.8) | 22.3 (3.8) | 24.3 (4.8) | 23.1 (3.9) | 24.9 (4.7) |
| ** Diameter | 30.2 (5.3) | 33.2 (7.6) | 29.1 (5.0) | 31.6 (6.1) | 30.2 (5.3) | 32.3 (6.0) |
| Maximum Betweenness Centrality | 0.63 (0.05) | 0.63 (0.06) | 0.63 (0.04) | 0.62 (0.05) | 0.63 (0.05) | 0.62 (0.03) |
| Leaf Fraction | 0.42 (0.03) | 0.41 (0.04) | 0.43 (0.04) | 0.42 (0.02) | 0.41 (0.03) | 0.4 (0.03) |
| * Degree Divergence | 1.13 (0.10) | 1.11 (0.10) | 1.17 (0.10) | 1.12 (0.07) | 1.12 (0.07) | 1.08 (0.08) |
|  |  |  |  |  |  |  |
| CNS Volumes and cortical thickness | | | | | | |
| Amygdale^1^ | 3202.5 (502.5) | 3203.2 (445.1) | 2937.6 (573.4) | 2768.2 (440.7) | 2790.6 (552.8) | 2773.6 (710.2) |
| Accumbens area^1^ | 843.2 (192.4) | 901.1 (169.0) | 760.0 (185.6) | 811.0 (164.3) | 792.0 (182.3) | 698.9 (206.9) |
| ^++^ Hippocampus^1^ | 7701.1 (1009.6) | 7660.4 (940.9) | 7271.7 (1159.5) | 7043.3 (975.6) | 6794.2 (1012.4) | 6489.8 (1182.8) |
| ^++^ Precuneus^2^ | 2.23 ( 0.12) | 2.31 ( 0.09) | 2.19 ( 0.16) | 2.16 ( 0.10) | 2.14 ( 0.14) | 2.09 ( 0.12) |
| ^+^ Entorhinal cortex^2^ | 3.20 ( 0.33) | 3.22 ( 0.28) | 3.21 ( 0.40) | 3.22 ( 0.40) | 3.00 ( 0.42) | 2.90 ( 0.49) |
| ^+^ Isthmus of the cingulate gyrus^2^ | 2.15 ( 0.14) | 2.25 ( 0.13) | 2.15 ( 0.16) | 2.09 ( 0.13) | 2.08 ( 0.14) | 2.03 ( 0.11) |
| Parahippocampal gyrus^2^ | 2.64 ( 0.24) | 2.68 ( 0.25) | 2.63 ( 0.22) | 2.66 ( 0.31) | 2.48 ( 0.22) | 2.58 ( 0.29) |
| Orbitofrontal cortex^2^ | 2.39 ( 0.10) | 2.39 ( 0.12) | 2.38 ( 0.17) | 2.33 ( 0.15) | 2.31 ( 0.13) | 2.30 ( 0.12) |
| ^+^ Anterior cingulate gyrus^2^ | 2.50 ( 0.16) | 2.48 ( 0.12) | 2.49 ( 0.20) | 2.52 ( 0.25) | 2.43 ( 0.18) | 2.36 ( 0.19) |
| ^+^ Fusiform gyrus^2^ | 2.60 ( 0.13) | 2.67 ( 0.14) | 2.61 ( 0.16) | 2.55 ( 0.18) | 2.51 ( 0.17) | 2.47 ( 0.18) |
| WMH^1^ | 3177.7 (4678.0) | 2777.1 (3800.8) | 4417.3 (6352.6) | 3195.1 (4652.4) | 3693.6 (3389.9) | 3247.8 (2477.5) |
|  |  |  |  |  |  |  |
| *Standard Deviations (SD) in brackets*  ^1^ volumes (mm3)  ^2^ thickness in mm  ** significant difference between depressed and non-depressed subjects after correction for multiple testng  * significant difference between depressed and non-depressed subjects at a nominal level (without correction)  ^++^ significant difference between MCI and non-MIC subjects after correction for multiple testing  ^+^ significant difference between MCI and non-MIC subjects at a nominal level (without correction) | | | | | |  |


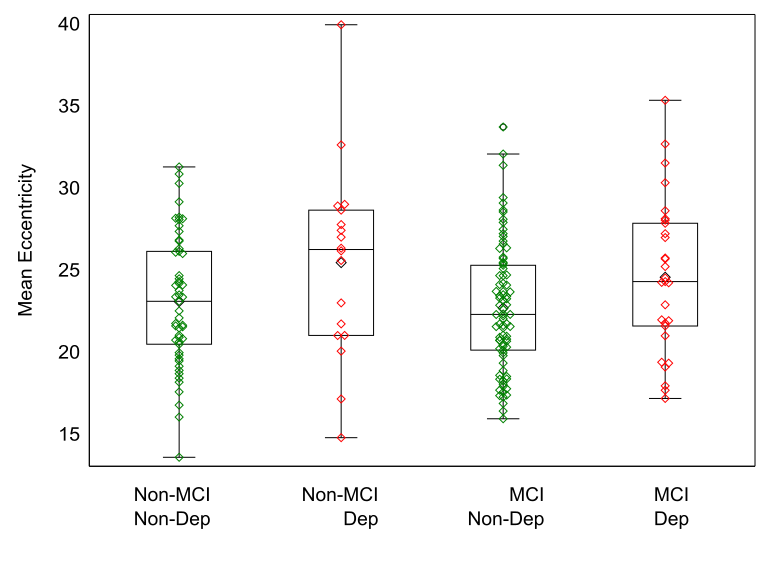


**Supplement Figure 1**

Mean Eccentricity in the study groups. DEP: subjects showing depressive symptoms; non-DEP: patients without depressive symptoms; MCI = Mild Cognitive Impairment; Non-MCI: subjects without MCI.

The display includes a box spanning the Q1-Q3 inter-quartile range, with a line drawn at the median value. A black diamond marks the mean value.


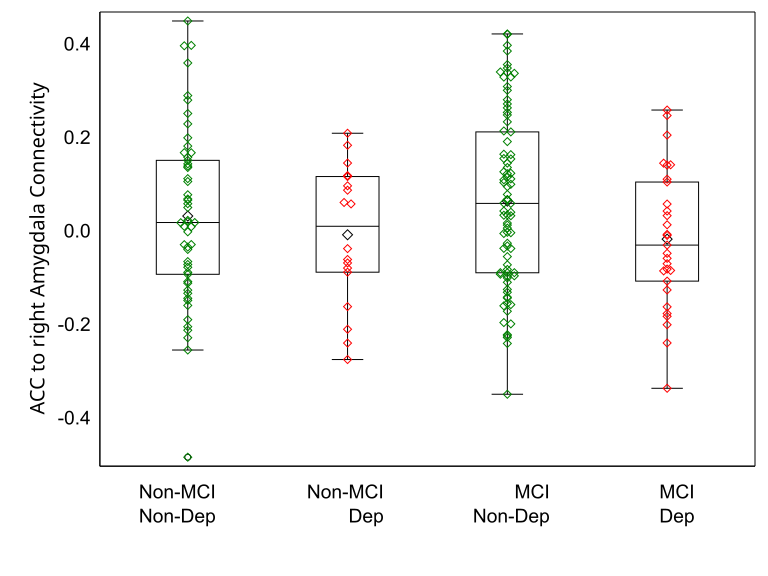


**Supplement Figure 2**

Anterior Cingulate Cortex (ACC) to right Amygdala functional connectivity in the Forntolimbic network. DEP: subjects showing depressive symptoms; non-DEP: patients without depressive symptoms; MCI = Mild Cognitive Impairment; Non-MCI: subjects without MCI.

The display includes a box spanning the Q1-Q3 inter-quartile range, with a line drawn at the median value. A black diamond marks the mean value.

**References**

1 Whitfield-Gabrieli S, Nieto-Castanon A. Conn: a functional connectivity toolbox for correlated and anticorrelated brain networks. *Brain Connect* 2012; **2**: 125–141.

2 Reuter M, Rosas HD, Fischl B. Highly accurate inverse consistent registration: a robust approach. *Neuroimage* 2010; **53**: 1181–1196.

3 Ségonne F, Dale AM, Busa E, Glessner M, Salat D, Hahn HK *et al.* A hybrid approach to the skull stripping problem in MRI. *Neuroimage* 2004; **22**: 1060–1075.

4 Fischl B, Sereno MI, Dale AM. Cortical surface-based analysis. II: Inflation, flattening, and a surface-based coordinate system. *Neuroimage* 1999; **9**: 195–207.

5 Dale AM, Fischl B, Sereno MI. Cortical surface-based analysis. I. Segmentation and surface reconstruction. *Neuroimage* 1999; **9**: 179–194.

6 Fischl B, Sereno MI, Tootell RB, Dale AM. High-resolution intersubject averaging and a coordinate system for the cortical surface. *Hum Brain Mapp* 1999; **8**: 272–284.

7 Fischl B, Salat DH, van der Kouwe AJW, Makris N, Ségonne F, Quinn BT *et al.* Sequence-independent segmentation of magnetic resonance images. *Neuroimage* 2004; **23 Suppl 1**: S69-84.
